# Healing of External Wounds1Read before the June meeting of the Missouri Valley Veterinary Medical Association.

**Published:** 1900-07

**Authors:** J. A. Sloan

**Affiliations:** Kansas City, Mo.


### HEALING OF EXTERNAL WOUNDS.[1]

By J. A. Sloan, M.D.V.,
KANSAS CITY, MO.

The particular phase of the subject to be discussed in this paper is that relating to the pathological processes in the healing of wounds and with no particular reference to their treatment. The discussion will also be principally confined to wounds of such a size and conditions as are healed by the visible formation of new tissue and less fully to those which heal by primary union. There are two reasons why this part of hypertrophy and regeneration of tissue is selected. First, because such wounds are of everyday occurrence, and therefore important; and, second, because the subject is too vast to discuss in its entirety.

It is said "that a wound is a recent solution of continuity of the living tissues induced by some mechanical cause." Some writers hold that all wounds are on the outside surface of the body, involving an abrasion of the skin, but this will hardly cover contused wounds. While external influence may hasten or retard the process of repair they will not be considered except when suppuration intervenes.

The processes of repair apparently vary according to the severity of the injury, while in fact they are practically similar except in extent and length of time necessary to recovery. The exceptions to this are two: First, those where there is such a trifling destruction of cells that simple regeneration of the essential surrounding cells is sufficient for repair. Secondly, where the wound is somewhat greater in extent, but still within the capacity of the connective tissue to repair by hyperplasia and regeneration without the intervention of embryonic and granulation tissues.

By healing is meant the removal of foreign material from a wound and the replacement of the destroyed or degenerated tissues by new tissue. Hyperplastic and regenerative growth of tissue is ushered in by cell-multiplication, by karyokinesis, and are inseparably associated with the phenomena of inflammation. In fact, so much so that it is impossible to consider one subject without involving the other. Proliferation occurs most readily when there is little inflammation present and

[1] Read before the June meeting of the Missouri Valley Veterinary Medical Association.

only a slight exudate, for the presence of any irritant retards the process by increasing the inflammation and exudation, and may lead to suppuration.

The ability to form new tissue lies in the proliferative faculty of the cells. These do not multiply under normal conditions, but when a wound is to be repaired some kinds of tissues proliferate with readiness and rapidity.

Hyperplasia and regeneration of tissue are by no means confined to the repair of wounds, for they always accompany productive or proliferative inflammation. It is found that in the connective tissues is a reserve force which is ready and convenient, whenever the tissues surrounding a wounded area, whether due to injury, degeneration, or necrosis, cannot repair the breach. If the injury is beyond the capability of the surrounding tissues to repair, the space is filled by connective neoplasia or scar-tissue. Because inflammation is always present in these processes it has been called proliferative inflammation, and it is present regardless of the presence or absence of foreign material. If proliferation is repeatedly interfered with, as, for instance, because of great irritation by pathogenic germs or external violence, the cells lose their power of regeneration, and an ulcer is the result. On the other hand, proliferation in a healthy patient may be so active and extensive that exuberant granulations appear with a considerable thickening of the adjoining parts. At this stage anything that will reduce inflammation will check granulation.

Wounds are divided according to cause into: Incised, punctured, lacerated, contused, etc. In veterinary practice external wounds are generally infected. This condition it is impossible to avoid because of septic surroundings and the impossibility in many cases of applying a bandage.

Wounds are said to heal by first intention, second intention, granulation, etc. The reparative process is much the same in all of these, for in all there has been destruction of tissues, and regeneration is necessary to repair. Therefore such a classification seems unnecessary except as a matter of convenience. There is a macroscopic difference in the healing of a wound whose edges are held in close apposition and whose changes are hidden from the eye and that of a large, open wound where the granulations can be plainly seen, but practically the means of repair are similar.

Healing by first intention occurs in slight septic wounds where there is a little destruction of tissues, in incised wounds and where the edges can be brought in close apposition. If there is no foreign material to be eliminated there should be an almost imperceptible amount of inflammation present and very little exudate to be absorbed or otherwise disposed of. Union is then hastened by plastic adhesion of opposing edges by means of the serous exudate. This adhesion plays a passive part in regeneration of wounds and, as repair progresses, is absorbed.

Adhesion has been mistaken for healing and desiccation of the exudate for the immediate formation of a scab. That this adhesion is not healing is proven in cases where pus-germs have been inclosed. In a day or two these germs, in presence of excellent nutrition, proper temperature, and moisture, multiply with the formation of pus, and the exudate softens and gives way. Immediate union is impossible. Adhesion may be sufficiently firm to require no bandage to keep the parts in apposition, but it is not healing.

The writer is convinced that plastic adhesion has taken place in wounds which have been said to have "healed in a few hours" or even in a "day or two" and not healing. This adhesion aids the reparative process by holding the edges of the wound in close apposition, preventing motion of these edges on each other and excluding foreign materials. The presence of any foreign material will prevent healing by primary union, because it becomes an irritant, setting up increased inflammation, with its attendant phenomena of emigration of leucocytes and formation of pus. Even extravasated blood if in appreciable quantities becomes a foreign body and an irritant, and furnishes a medium for the propagation of bacteria. In no instance is it possible for this extravasated blood to assist in the process of repair, and it is a common mistake for veterinary surgeons to bind up a wound before hemorrhage ceases. The blood, too, may form a clot in the wound, prevent all possible contact of opposing edges, and thus give a cavity which later must be filled by granulation.

When a small, aseptic, incised wound is closed by sutures an exudate takes place from the suture opening. This is because the sutures have made a small, open wound with destruction of more or less tissue. It is nicely illustrated by a neat operation of plantar-neurectomy. If the injury is very slight new cells are soon formed to replace those destroyed, with little or no change in structure, and is the nearest possible approach to immediate union. In this case, the earliest signs of proliferation may be seen in about eight hours after infliction of the injury, and very readily seen after twenty-four to forty-eight hours. In wounds of a somewhat larger size there is infiltration of the surrounding tissues, exudate upon the cut surface, and later proliferation of connective tissue from the sound tissue through the infiltrated area and into the wound, which finally firmly binds the edges and constitutes healing by primary union. Proliferation of epithelium completes the process.

As an aid to primary union these steps should be observed: First, arrest and prevent hemorrhage; second, to remove all foreign substances; third, apply suitable bandages, both to keep the parts in close approximation and also to prevent the entrance of pathogenic germs. About the shortest possible time for these wounds to heal under the most favorable circumstances is seven to eight days.

Healing by granulation or so-called second intention is seen in wounds where the changes are visible to the unaided eye and where there has been extensive destruction of tissue. This process consists of three stages: First, the formation of a deteriorated or embryonic tissue, which develops into, second, granulation tissue, and third, completes the repair by a further change into cicatricial tissue. This process is entirely competent to repair any wound or to make an uninterrupted continuity of tissues. The repair is not of normal tissue, and there is a loss of function because of the intervention and usually the preponderance of the connective tissue. The normal tissues do not possess the ability to close up the wound.

Embryonic tissue consists largely of cells and bloodvessels, and is never found except where it is beyond the ability of the surrounding tissues to repair the injury. The cells are mostly small, rounded in shape, and infiltrated with leucocytes—mono- and polynuclear—which have emigrated from the circulation. The number of leucocytes determine the intensity of the inflammation present, and conversely the rapidity of recovery. The cells of embryonic tissue have been called proliferative or formative cells, because they give rise to the future tissue. In the early stage of its development it depends upon the tissue from which it sprung for its support, but later bloodvessels quickly develop for its sustenance and growth.

The bloodvessels proliferate by a process of budding or sprouting from the neighboring uninjured vessels, and develop rapidly with the formation of numerous loops which give a reddish color to the tissue. About each loop is a collection of cells which give to the tissue a beady appearance. The rich vascular supply favors a rapid cell-growth and an abundant proliferation. From connective-tissue cells fibroblasts are formed, which change into branch and spindle-shaped cells and later lie closely together, forming the bundles of fibres which constitute the cicatricial tissue. Granulation tissue is the next stage, and is developed from the embryonic by the formation of cellular elements similar to the parent tissues. Part of the cells are in large or hypertrophied tissue cells which undergo division and enlargement indefinitely, depending on the extent of the injury and favorable termination of the whole process. When it develops into granular masses it is popularly known as "proud flesh" and is a source of uneasiness to the layman. While it is confined to the cavity which it attempts to fill, or does not project beyond the edge of the wound, no interference is necessary, as it is the proper and only means of repair. This tissue is also very vascular, and is very easily injured because it has no protective covering.

The third stage is the development of granulation into cicatricial or scar-tissue. This is accomplished through the formation of branched and spindle-shaped fibroblasts which unite into bundles. Proliferation of the epithelium has followed the filling up of the cavity as far as possible, and when it ceases the rest of the new tissue is covered by a dense layer of cicatricial tissue or "scar." At this stage the scar is reddish in color, smooth, devoid of hair or glands of the skin and tender to the touch. With the formation of cicatricial tissue the reparative process is completed, although changes still occur. Contraction begins, causing atrophy of all its tissues. Bloodvessels, nerves, and connective tissue diminish, and consequently the size of the cicatrix, and may lead to deformity and partial loss of function. It becomes of a dense white appearance and approaches more and more to the tissues from which it springs. These latter changes may spread over a period of several months.

At the risk of being tedious I will refer quite briefly to the important phenomenon of phagocytosis, by means of which pathogenic germs are destroyed. It is held by some pathologists that phagocytosis begins with the emigration of leucocytes, but later is actively carried on by the phagocytes and fibroblasts or cells of connective tissue origin. When only a few bacteria are present they may become a prey to the leucocytes, and if no other germs gain access to the wound infection will not follow. There is little doubt but that this process plays an important part in the healing of wounds by preventing in part or entirely the multiplication of bacteria. This phenomenon is accomplished by the amœboid movement of the leucocytes, each cell inclosing a germ. The fibroblasts may close half a dozen cells, as the leucocytes do the cause of infection or any other cells, living or inert. The bacterium may be retained until destroyed or carried into the lymphatic system and deposited in a lymphatic gland. Sometimes the bacterium is too virulent and destroys the leucocytes, and so obtains its freedom. As phagocytosis tends to limit the multiplication of pathogenic germs, and in that way prevents an increase in extent and intensifies inflammation, so its effect on regeneration will be appreciated when we remember that wounds heal most quickly when there is no infection and but little inflammation present.

